# A combined use of silver pretreatment and impregnation with consequent Nissl staining for cortex and striatum architectonics study

**DOI:** 10.3389/fnana.2022.940993

**Published:** 2022-10-14

**Authors:** Gennadii Piavchenko, Vladislav Soldatov, Artem Venediktov, Natalia Kartashkina, Natalia Novikova, Marina Gorbunova, Tatiana Boronikhina, Alexander Yatskovskiy, Igor Meglinski, Sergey Kuznetsov

**Affiliations:** ^1^Department of Histology, Cytology, and Embryology, I.M. Sechenov First Moscow State Medical University (Sechenov University), Moscow, Russia; ^2^Department of Pharmacology and Clinical Pharmacology, Belgorod National Research University, Belgorod, Russia; ^3^Laboratory of Pathophysiology, Institute of Experimental Medicine, Saint Petersburg, Russia; ^4^Department of Histology, Cytology, and Embryology, Orel State University named after I.S. Turgenev, Orel, Russia; ^5^Opto-Electronics and Measurement Techniques, Faculty of Information and Electrical Engineering, University of Oulu, Oulu, Finland; ^6^College of Engineering and Applied Science, Aston University, Birmingham, United Kingdom

**Keywords:** staining combination, Nissl staining, silver impregnation, protoplasmic astrocytes, artificial pseudostaining

## Abstract

Despite a rapid growth in the application of modern techniques for visualization studies in life sciences, the classical methods of histological examination are yet to be outdated. Herein, we introduce a new approach that involves combining silver nitrate pretreatment and impregnation with consequent Nissl (cresyl violet) staining for cortex and striatum architectonics study on the same microscopy slide. The developed approach of hybrid staining provides a high-quality visualization of cellular and subcellular structures, including impregnated neurons (about 10%), Nissl-stained neurons (all the remaining ones), and astrocytes, as well as chromatophilic substances, nucleoli, and neuropil in paraffin sections. We provide a comparative study of the neuronal architectonics in both the motor cortex and striatum based on the differences in their tinctorial properties. In addition to a comparative study of the neuronal architectonics in both the motor cortex and striatum, the traditional methods to stain the cortex (motor and piriform) and the striatum are considered. The proposed staining approach compiles the routine conventional methods for thin sections, expanding avenues for more advanced examination of neurons, blood–brain barrier components, and fibers both under normal and pathological conditions. One of the main hallmarks of our method is the ability to detect changes in the number of glial cells. The results of astrocyte visualization in the motor cortex obtained by the developed technique agree well with the alternative studies by glial fibrillary acidic protein (GFAP) immunohistochemical reaction. The presented approach of combined staining has great potential in current histological practice, in particular for the evaluation of several neurological disorders in clinical, pre-clinical, or neurobiological animal studies.

## Introduction

Nowadays, staining methods are used extensively in the current practice of identification and assessment of nervous tissue malformation (Kirik et al., 2013; Braak et al., 2018; Czechowska et al., 2019; Vints et al., 2019). Nevertheless, while immunohistochemistry and electronic microscopy improve significantly the power of visualization techniques enhancing informativity and performance of biomedical diagnostics, the potential of classical staining methods has not yet been exhausted. New methods have been suggested to enhance the accuracy of silver impregnation and Nissl staining, and to advance the methodology of nervous tissue staining (Czechowska et al., 2019; Vints et al., 2019). Various stains are widely known and available for identifying neurons, macroglia, and microglia, as well as certain subcellular structures and even neurobiological processes. A relevant challenge for modern morphology is to find such a combination of easy-to-access methods which would enable a comprehensive analysis of tissue structure due to original tinctorial features (Braak et al., 2018). The routine morphological methods, such as hematoxylin-eosin, cresyl violet, and toluidine blue, reveal successful staining of structures and previously required immunohistochemical techniques (Korzhevskii et al., 2006; Kirik et al., 2013).

For a long time, the silvering technique has been employed to examine the morphology of pyramidal neurons and their processes, as well as to examine glial cells, neuropil, and nerve fibers (Jacobs et al., 2018; Joseph et al., 2019). Silver impregnation techniques are able to identify only 5–10% of all neurons, while the rest of the cells remain non-impregnated. In contrast, the Nissl methods allow staining of the abundant number of neurons and also detect a synthetic activity of the cells, which is marked by the amount of chromatophilic substance, i.e., ribosomal ribonucleic acid in the rough endoplasmic reticulum and polyribosomes (Huber, 1966; P’yavchenko et al., 2016; Piavchenko et al., 2017). The combined use of classic Nissl and Golgi methods to stain the nervous tissue has been proposed earlier (Glaser and Van der Loos, 1981; Pilati et al., 2008; Kassem et al., 2013), but these show several serious drawbacks both in methodical and practical aspects. These techniques are not able to visualize architectonics of the cerebral cortex and striatum, and, therefore, are suitable only for the studies of cytoarchitectonics of the cerebellum and some of the brain stem nuclei. With regard to astrocyte staining, the cells are difficult to detect in gray matter with traditional methods. In addition, due to typical negative reactions in astrocytes, a careful adjustment in the dilution of a primary antibody to glial fibrillary acidic protein is required in immunohistochemistry (Walz and Lang, 1998).

In the current study, the architectonics of the cerebral cortex and striatum of rats are examined on the same microscopy slide by employing a combined approach of silver nitrate pretreatment and impregnation with the consequent Nissl (cresyl violet) staining.

## Materials and methods

### Animals

One-month-old *Sprague Dawley* male rats (*n* = 24), obtained from the certified vivarium “Pushchino” (Institute of Bioorganic Chemistry of Russian Academy of Science), are used in the current study. Initially, the animals are placed in quarantine for 2 weeks upon enrollment. According to recommendations of the Organization for Economic Co-operation and Development principles of Good Laboratory Practice, all the animals are kept in controlled environmental conditions (temperature of 20 to 26°C, relative humidity of 30 to 70%, bacterial purity, 10 air changes per hour, and a 12-h day/light cycle) (OECD, 1998). Animals are regularly examined by a veterinary practitioner and those that were found to develop marked deviations are excluded from the experiment. All animal procedures are performed in accordance with the requirements of Directive 2010/63/EU, which is the European Union (EU) legislation “on the protection of animals used for scientific purposes,” and approved by Sechenov First Moscow University local ethics committee (protocol number 03-19).

### Anesthesia, fixation, and brain autopsy

The animals are anesthetized with intramuscular injections of Zolazepam + Tiletamin (Zoletil^®^ 100, Virbac, Carros, France) at the dose of 50 mg/kg. After that, intracardiac perfusion into the left ventricle of the heart with isotonic sodium chloride solution and heparin followed by a vital fixation in a 10% solution of neutral formalin in phosphate-buffered saline is performed. The brain of the perfused animals is consequently removed and fixed with a new portion of the aforementioned fixative substances.

### Pretreatment by silver nitrate solution

The fixed brain tissue is incubated in 10% silver nitrate water solution in a dark place at room temperature for 3–4 days. Then, the examined tissue sample is dehydrated in ascending ethyl alcohol solution concentrations (50, 70, 96, and 100% for 2 h in each) followed by two portions of chloroform (2 h in each) and two portions of paraffin (in an incubator at the temperature of 60°C for 2 h in each) with a tissue embedding in paraffin (Garman et al., 2016).

### Slicing

The paraffin-embedded brain tissue blocks are sliced into 5-μm thin and 20-μm thick coronal sections with an HM 430 Sliding Microtome (Thermo Scientific, Waltham, MA, USA) equipped with disposable blades.

### Impregnation and staining

To perform combined nervous tissue staining, the sliced sections are deparaffinized in two portions of xylene with 5 min in each, and treated consequently with a series of ethyl alcohol solutions (decreasing concentrations: 96, 70, 50, and 30% for 5 min in each) and finally with distilled water for 2 min).

The deparaffinized tissue sections are incubated in a glass container at the temperature of 40°C for 5–8 min. The incubation is performed in the aqueous solution of 10% silver nitrate, after concentrated ammonia (30%) is added drop-by-drop until the silver is settled, and the mixture within the container becomes transparent.

Then, the slides are placed in the solution containing formalin (20 ml), citric acid (0.5 g), concentrated nitric acid (2 drops), and distilled water (up to 100 ml) mixed with concentrated ammonium hydroxide in equal amounts (diluted with distilled water in the ratio of 1:4 for 40–60 s). Further, the slides are submerged for 1 min in a 1% solution of ammonium hydroxide.

After washing in distilled water, the slides are placed in a 10% sodium thiosulfate solution for 1 min, washed again, and stored in distilled water for 5 min. Later on, the slides are incubated in a 1% cresyl violet solution in acetate buffer for 15–20 min at a temperature of 60°C. Finally, they are rehydrated with ethyl alcohol at ascending concentrations (30, 50, 70, and 96%) for 5 min each, clarified in two portions of xylene for 5 min each, and mounted on the coverslips.

### Structure identification and study of architectonics

The prepared tissue slides are studied under the Axio Imager 2 microscope (Carl Zeiss, Jena, Germany), whereas image acquisition is performed by using an AxioCam camera with further processing in the ZEN image analytic system.

### Immunohistochemical anti-GFAP staining

Anti-GFAP immunohistochemical reaction is carried out using polyclonal antibodies against GFAP (ab68428, Abcam, Cambridge, UK, 1:200 dilution), secondary antibodies (Goat anti-rabbit IgG ab97051, UK, 1:1,000 dilution), followed by peroxidase detection by adding diaminobenzidine chromogen (ab64238) on the paraffin-embedded material. The sections of tissue samples are then counterstained with Mayer’s hematoxylin, washed in water, dehydrated, and mounted.

### Measurements and data analysis

To determine the number of cells and chromatophilic substance area per cell, six independent fields of view from each specimen (two sections per animal and six animals in every group) are examined with an ocular of 20x and an objective of 40x resolution. The studies of architectonics of the third layer in the motor cortex and striatum are performed according to the stereotaxic atlas (Paxinos and Watson, 2014). In order to compare different staining approaches and to optimize each step to the new one, the brain sections are treated separately with silver impregnation, with cresyl violet Nissl staining, and with silver impregnation without pretreating in silver solution.

The Origin 2021 software (OriginLab Corporation, Northampton, MA, USA) is used for a comparative statistical analysis (Prentice, 2007; Yan et al., 2017) of the data obtained in the regions of the motor cortex, piriform cortex, and striatum. The Kolmogorov–Smirnov criterion is used to test deviation from the normal distribution, and the median and interquartile percentile (Me, 25L; 75U) is evaluated with the Mann–Whitney U-criterion. The probability of obtaining a real-valued test statistic is found to be ∼ 95% with a statistical significance *p* ≤ 0.05.

The transfer of 2D images of histological sections to the 3D surface format is performed in MATLAB (MathWorks, Natick, MA, USA). The identified structures, such as “impregnated neurons,” “cresyl violet-stained neurons,” “intercellular substance,” “protoplasmic astrocytes,” and “vessels,” are highlighted by a histologist on the image of the section of a tissue sample.

## Results

**FIGURE 1:**
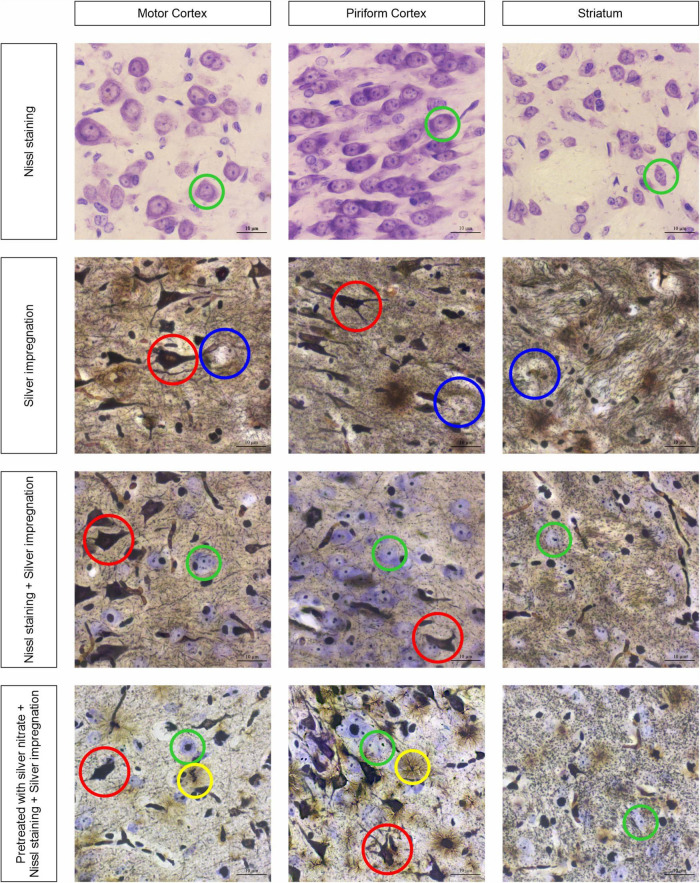
Three brain regions, the motor cortex, the piriform cortex, and the striatum, stained with four different approaches: neurons with cresyl violet (green circles); neurons with silver nitrate (red circles); non-impregnated neurons (blue circles); and astrocytes (yellow circles). Ob. 63x, oc. 20x, scale bar: 10 μm.

Figure 1 shows the sections of tissue samples stained by different methods: Nissl with cresyl violet, silver impregnation, a combination of silver impregnation and the Nissl staining, and a new approach with combined silver pretreatment and impregnation with consequent Nissl staining. Three brain regions are selected to show tinctorial properties of the nervous tissue, namely, the motor cortex that takes part in the motor activity regulation (Donoghue and Wise, 1982), the well-developed piriform cortex that is responsible for the olfactory sense in rats (Wilson, 2001), and the striatum participating in the regulation of motor and reward behavior (Brown and Sharp, 1995) and establishing close contact with the motor cortex (Steiner and Kitai, 2000).

The obtained results show that while the sections are stained with cresyl violet only (Nissl staining), the neurons of different sizes become visible with reasonably high resolution (see Figure 1, first row of images). The largest of them are found in the motor cortex, whereas in the piriform cortex and striatum they seem to be smaller (green circles). In addition, the chromatophilic substance, nucleus, and nucleoli are clearly observed. As soon as the tissue sections are impregnated with silver nitrate (see Figure 1, second row of images), a number of totally impregnated neurons of different sizes and forms appear to be visible in cortical regions, whereas sporadic neurons with processes are seen in the striatum (red circle). The light-brown outlines show almost not impregnated neurons (blue circles) in the second row of images and transversally oriented bands of nerve fibers in the striatum. When the combined use of two approaches is applied, both types of neurons are found: totally impregnated and stained with cresyl violet (see Figure 1, third row of images; red and green circles, respectively). The nerve fibers in the striatum are similar to those observed following treatment with isolated silver nitrate, whereas the neuropil is found to have a light brown color in the striatum. The inclusions mentioned above can be clearly observed with a combined use of silver pretreatment and impregnation with consequent Nissl staining (see Figure 1, fourth row of images), including totally impregnated neurons (red circles), cresyl violet-stained neurons (green circles), and astrocytes (yellow circles).

**FIGURE 2:**
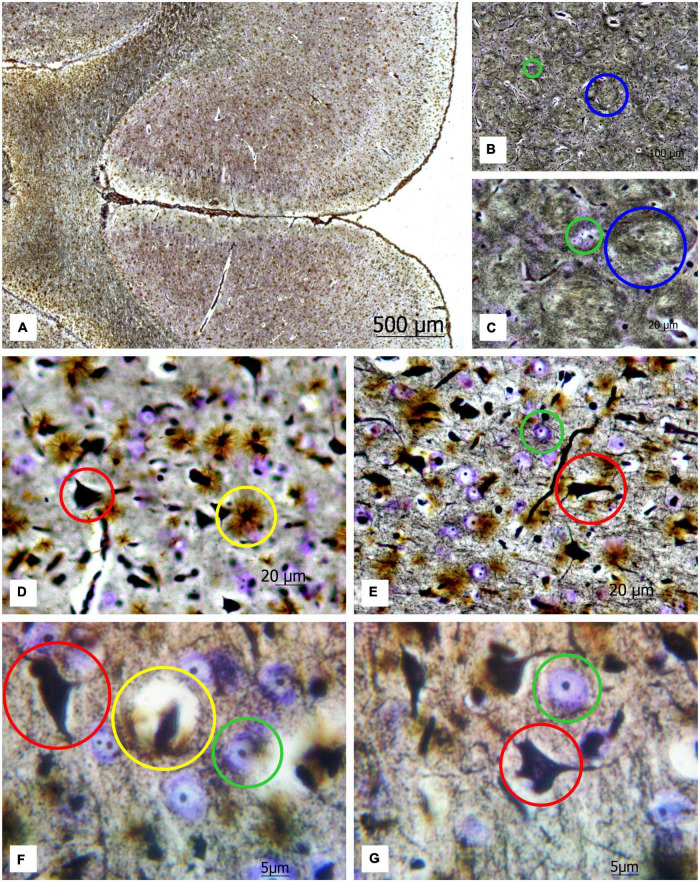
**(A)** Image of a rat brain after combined staining. Ob. 2.5x, oc. 20x; **(B)** transversely oriented nerve fibers (blue circles) and **(C)** neurons stained by the Nissl method (green circles) highlighted in the image of the rat striatum. Ob. 40x, oc. 20x; **(D)** protoplasmic astrocytes (yellow circles), impregnated neurons (red circles); **(E)** neurons stained with cresyl violet (green circles) highlighted in the image of the rat motor cortex. Ob. 40x, oc. 20x. **(F,G)** protoplasmic astrocytes (yellow circle), impregnated neurons (red circle), and neurons stained with cresyl violet (green circle) highlighted in the image of rat motor cortex. Ob. 100x, oc. 20x.

Applying a new hybrid staining approach for the assessment of the number and size of neurons and glial cells in the architectonics of the cortex and striatum (Figure 2A), a large number of transversely oriented nerve fibers in the striatum are observed, along with dispersed focal neurons between them, which are stained predominantly with cresyl violet (see Figures 2B,C). One can also detect some solitary impregnated neurons.

This method enables to reveal chromatophilic substance, nucleoli, neuropil (Figures 2D,E) as well as the protoplasmic astrocytes (Figures 2F,G). As one can see in the sections of the motor cortex in rats, the cresyl violet-stained neurons with chromatophilic substances and a great number of totally impregnated neurons are observed. The rounded structures of small size (nucleoli) in the nuclei of neurons, stained by the Nissl method, neuropil, and the processes of various thicknesses projecting from the impregnated neurons in different directions are clearly observed.

**FIGURE 3:**
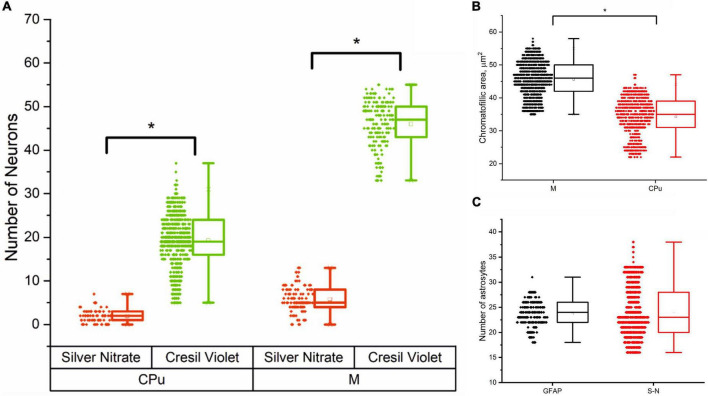
Cells morphometry in the motor cortex (M) and striatum (CPu) in male rats, Me (25L; 75U). **(A)** Number of silver nitrate impregnated and cresyl violet-stained neurons in the striatum (CPu) and motor cortex (M); **(B)** Average chromatophilic substance area per cell, μm^2^ in the motor cortex (M) and striatum (CPu); **(C)** Number of silver nitrate impregnated (S-N) and GFAP-positive (GFAP) astrocytes in the motor cortex.

Figure 3 presents an analysis of the number of neurons and astrocytes and their functional activity in the motor cortex and striatum.

**FIGURE 4:**
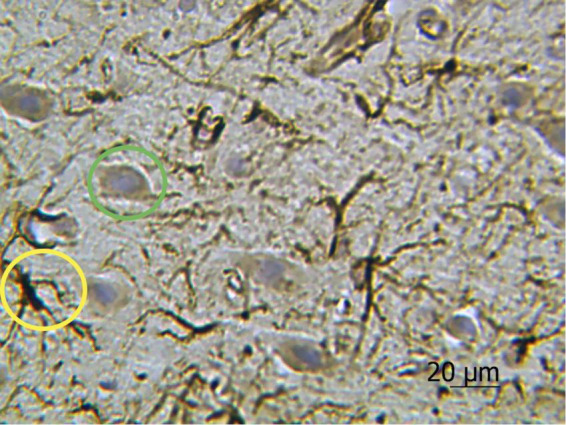
Rat motor cortex treated with anti-GFAP immunohistochemical reaction with the highlighted examples of protoplasmic astrocytes (yellow circle) and neurons stained with hematoxylin (green circle). Ob. 40x, oc. 20x.

The results of cell morphometry analysis presented in Figure 3A suggest that just a minor part of neurons (∼10% of the total amount) are impregnated with silver nitrate. The results also show that the number of neurons and the average area of chromatophilic substance in the striatum is notably less than in the motor cortex (see Figures 3A,B). The number of neurons in the motor cortex is about two times higher when compared to the number of astrocytes in the same region (see Figures 3A,C). A comparative analysis shows that the results independently obtained with two staining methods, that is, anti-GFAP immunohistochemical reaction (Figure 4) and the new hybrid staining approach, agree well with each other with no significant statistical difference (see Figure 3C). Moreover, the acquired images show the presence of complex patterns that most likely can be identified in the central nervous system by the new hybrid staining method.

**FIGURE 5:**
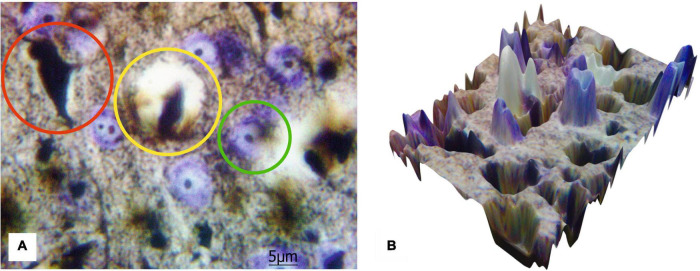
The 2D image of rat motor cortex **(A)** with the highlighted protoplasmic astrocyte and the vessel (yellow circle), impregnated neurons (red circle), and neurons stained with cresyl violet (green circle). Ob. 100x, oc. 20x; **(B)** corresponding 3D surface for the 2D histological image of the same histological section.

Figure 5 shows the results of the transfer of 2D images of histological sections to the 3D surface format performed by MATLAB for a more progressive quantitative analysis. The presented results emphasize an opportunity to distinguish the components of blood–brain barrier and technical capacity to create 3D data set suitable for further quantitative analysis with Artificial Intelligence (AI) processing utilizing Machine Learning and Deep Learning algorithms.

**FIGURE 6:**
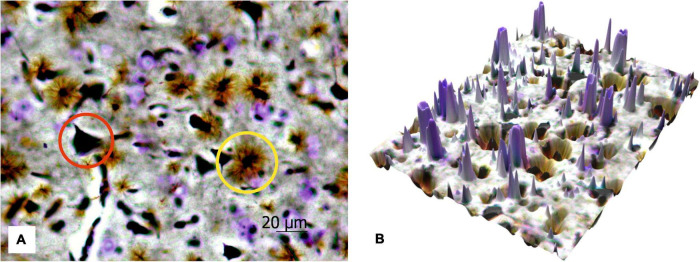
The 2D image of rat motor cortex **(A).** The protoplasmic astrocytes (yellow circle) and impregnated neurons (red circle) are in focus, and the neurons stained with cresyl violet are sharp. Ob. 40x, oc. 20x; **(B)** corresponding 3D surface for the 2D histological image of the same histological section.

Figure 6 shows the protoplasmic astrocytes, observed in focus, as well as both short and highly branching processes, whose bodies are black similar to the bodies of the neurons. The results also display that the number of astrocytes is about two times less than all the neurons (both cresyl violet-stained and silver-impregnated). The transfer of the 2D histological images to 3D surface format (see Figure 6B) with MATLAB allows seeing all the structures in the 3D landscape that can be further used for stand-alone quantitative analysis with AI technologies.

## Discussion

Despite the fact that new modern imaging techniques are growing fast and widely used in histopathology studies, the conventional histostaining methods remain the “gold standard” for examining the nervous tissue both in a normal state and in pathology. Nissl staining approaches are used frequently in combination with more recent approaches, providing reference and quality control. Nevertheless, the methods compiling Nissl staining with immunohistochemistry are still able to supply new data, especially in neurochemistry (Mansour et al., 2019). The use of Nissl staining with immunohistochemistry and electron microscopy is also in high demand (Chiarotto et al., 2019). For example, it has been demonstrated that such techniques may be useful for astrocyte detection (Korzhevskii and Otellin, 2005).

The neuron silvering methods, proposed by Ramón y Cajal and Bielschowsky, also provide better visualization of a list of morphological features (Bielschowsky, 1904; Ramón y Cajal, 1904). Such silver staining methods use argyrophilic properties of cells, especially those which are characteristic of nucleoli (Trerè, 2000; Rivas-Manzano et al., 2021). Combining classical silver staining methods and immunohistochemistry together provides an opportunity for the quantitative assessment of tissue sections (Ahmad et al., 2019; Preziosa et al., 2019). Silver staining methods, e.g., the one described by Bielschowsky, have proved their efficacy in combination with a single- or double-immunofluorescence of the same section for detecting molecular changes (Bielschowsky, 1904; Segura-Anaya et al., 2018).

According to the literature, silver nitrate impregnation occurs due to the precipitation of metallic silver on a protein substrate (Pearse, 1960), especially on electron-rich groups (Chernousova and Epple, 2013). The pretreatment of samples with silver nitrate prior to ammoniacal solution enhances the precipitation process (Uchihara, 2007). After the formation of silver-ammonium ion complexes, silver visualization is achieved *via* aldehyde processing in optimal buffer conditions so that colloidal silver is released which now binds the most argyrophilic sites of cells. Thus, the neuronal metabolic activity seems to influence the number of stained neurons; this might explain the differences in the quantitative results in silver impregnation and Nissl staining methods. Comparative analysis of morphological patterns obtained for the cortex and striatum tissue sections, performed in the current study, show different numbers of silver nitrate-impregnated and cresyl violet-stained neurons, with a similar ratio between them. Therefore, we conclude that protein synthesis occurring in the neurons during their functioning might contribute to the silver reduction.

In order to achieve a stable silver staining process, a new buffer is created, using a few different compositions re-capitulating a cross between buffer for Nissl staining and buffer for silver nitrate impregnation. Being comprehensively tested, the final buffer solution is selected for further research. As the fast impregnation methods have the disadvantage of frequent color fade, the buffer composition is preferred among other possible combinations while developing staining due to the long period of stain stability (at least 4 years).

The approach presented in the current study is used to observe two types of neurons and compare their amounts in both cortex and basal ganglia with a number of astrocytes. The ability to reveal astrocytes is one of the most remarkable points of the novel method. Unfortunately, traditional staining methods are not efficient for staining astrocytes. Nowadays, three main IHC markers are used to achieve the purpose (Zhang et al., 2019); however, at this point, one can meet additional problems. It seems that the GFAP method does not reveal a large number of astrocytes (Walz and Lang, 1998), although in the comparative analysis of astrocyte numbers (Figure 3C), we have found it to be almost equal to our staining technique. S 100 looks like an appropriate marker, but it is not specific (Rickmann and Wolff, 1995). Finally, monoclonal antibodies to NDRG2 demonstrate a higher level of specificity; at the same time, this latter method is often weaker than GFAP staining (Flügge et al., 2014).

Some staining procedures, e.g., the Golgi-Cox staining method (Zhong et al., 2019), show good results in the visualization of dendritic spines. Such techniques use pretreatment in the solution of potassium chromate and dichromate with mercuric chloride, while in our method, we use a 10% silver nitrate solution for the pretreatment. This may be one of the main reasons for obtaining different staining results. Golgi-Cox staining requires thick sections (at least 100–200 μm) to be prepared and allows structural analysis of the dendrite surface. As presented combined staining approach is suitable for the assessment of neurons and astrocytes, we suppose that thin slices of 5 to 20 μm is the best option, although staining of thicker slices (50–150 μm) is also possible.

Since histopathological verification of human brain disorders usually relies on immunostaining techniques, the major application of the method is in animal research and pre-clinical trials. Therefore, there is a high potential for the developed method to find straightforward application in the current practice of clinical diagnosis of astrocytoma and epilepsy course prediction. Moreover, the method could be widely implicated in animal studies of neuropharmacology (Gorshkov et al., 2018; Piavchenko et al., 2021), neurodevelopment (Shevelkin et al., 2020; Singha et al., 2021), encephalomyelitis (Cammer et al., 1990), neurodegeneration (Leyns and Holtzman, 2017; Habib et al., 2020), stroke (Laterza et al., 2018), traumatic brain injury (Mannix et al., 2014), neuroprotection (Djebaili et al., 2005; Rossi et al., 2013), neurotoxicity (Demir et al., 2017), and other research studies which focus on the glial response. To date, the most prevalent method for the detection of glial response is immunostaining with antibodies to glial fibrillary acidic protein (GFAP). Glial fibrillary acidic protein (GFAP) is an intermediate filament-III protein uniquely found in the astrocytes of the central nervous system, non-myelinating Schwann cells in the peripheral nervous system, and enteric glial cells (Yang and Wang, 2015). The astrocytes of gray matter are difficult to observe with traditional methods, and IHC is also a tricky method because dilution of the primary antibody to GFAP needs careful adjustment. Even though GFAP-immunostaining is an extremely efficient and helpful method, still continuing to be updated and improved (Shin et al., 2009; Jurga et al., 2021), we consider our method as its simplistic alternative. Additionally, the method enables to avoid the restrictions related to interspecies differences in the reactivity of antibodies y, so the same protocol can be applied in the comparative studies of the neuroanatomy of various species.

We also conclude that the combined hybrid staining method retains the advantages of three other aforementioned procedures. It allows visualization of all the structures within the same slide and in the form of a 3D landscape suitable for further AI-based processing. Thus, the approach should provide new opportunities for quantitative analysis in the different brain regions, as well as to make us understand possible interactions between two types of stained neurons and astrocytes of the same section.

## Conclusion

The presented novel methodology enables us to combine the existing methods of the Nissl staining (cresyl violet) and silver nitrate impregnation in such a way that two types of neurons and astrocytes are detected in thin paraffin sections. The presented approach is able to demarcate the impregnated neurons (about 10%), Nissl-stained neurons (the rest of them), protoplasmic astrocytes, chromatophilic substance, nucleoli, and neuropil. Therefore, it is appropriate for a more advanced quantitative comparative analysis of architectonics in the brain structures which differ by tinctorial properties.

The developed hybrid staining technique permits the integration of various staining procedures within one section. We expect that it reveals neurons, blood–brain barrier components, and fibers. The method has a strong potential to be implemented in current clinical practice instead of immunohistochemistry for a routine biomedical inspection of nervous tissue both in normal and pathological conditions. The most evident applications include a severity assessment and prognosis of epilepsy (for surgically treated patients), relatively low-cost diagnostics test for anaplastic astrocytoma, as well as experimental assessment of acute cerebral ischemia provoked by cardiac cessation or respiratory arrest.

## Patents

Based on the research findings, we have obtained a patent for the invention: Pyavchenko (Piavchenko), G.A.; Dutta, P.; Novikova, N.S.; Nozdrin, V.I.; Method of Simultaneous Detection of Neurons and Astrocytes on Histological Preparations of Nervous Tissue. Official Bulletin of the Federal Service for Intellectual Property (RusPatent) 2018, 25, 2666256.

## Data availability statement

The original contributions presented in this study are included in the article/supplementary material, further inquiries can be directed to the corresponding authors.

## Ethics statement

The animal study was reviewed and approved by Institutional Ethics Committee of Sechenov University, Sechenov University, Moscow, Russia.

## Author contributions

GP: idea and stable protocol development. GP, VS, AV, NK, AY, and IM: writing. GP, NN, MG, TB, and AY: staining and analysis. IM and SK: supervision. All authors have read and agreed to the published version of the manuscript.
